# Effects of Human LAV-BPIFB4 Gene Therapy on the Epigenetic Clock and Health of Aged Mice

**DOI:** 10.3390/ijms24076464

**Published:** 2023-03-30

**Authors:** Maria Elisa Giuliani, Veronica Barbi, Giorgia Bigossi, Serena Marcozzi, Robertina Giacconi, Maurizio Cardelli, Francesco Piacenza, Fiorenza Orlando, Elena Ciaglia, Monica Cattaneo, Alessia Mongelli, Carlo Gaetano, Mauro Provinciali, Annibale Alessandro Puca, Marco Malavolta

**Affiliations:** 1 Advanced Technology Center for Aging Research, IRCCS INRCA, 60121 Ancona, Italy; 2 Laboratory of Epigenetics, Istituti Clinici Scientifici Maugeri IRCCS, Via Maugeri 10, 27100 Pavia, Italy; 3 Experimental Animal Models for Aging Unit, Scientific Technological Area, IRCCS INRCA, 60015 Falconara Marittima, Italy; 4 Department of Medicine, Surgery and Dentistry “Scuola Medica Salernitana”, University of Salerno, Via Salvatore Allende, 84081 Baronissi, Italy; 5 Cardiovascular Research Unit, IRCCS MultiMedica, 20138 Milan, Italy

**Keywords:** aging, epigenetic clock, frailty

## Abstract

The homozygous genotype of the Longevity-Associated Variant (LAV) in Bactericidal/Permeability-Increasing Fold-Containing Family B member 4 (BPIFB4) is enriched in long-living individuals of three independent populations and its genetic transfer in C57BL/6J mice showed a delay in frailty progression and improvement of several biomarkers of aging and multiple aspects of health. The C57BL/6J strain is a suitable model for studying therapies aimed at extending healthy aging and longevity due to its relatively short lifespan and the availability of aging biomarkers. Epigenetic clocks based on DNA methylation profiles are reliable molecular biomarkers of aging, while frailty measurement tools are used to evaluate overall health during aging. In this study, we show that the systemic gene transfer of LAV-BPIFB4 in aged C57BL/6J mice was associated with a significant reduction in the epigenetic clock-based biological age, as measured by a three CpG clock method. Furthermore, LAV-BPIFB4 gene transfer resulted in an improvement of the Vitality Score with a reduction in the Frailty Index. These findings further support the use of LAV-BPIFB4 gene therapy to induce beneficial effects on epigenetic mechanisms associated with aging and frailty in aged mice, with potential implications for future therapies to prevent frailty in humans.

## 1. Introduction

Naturally aged mice represent an excellent mammalian model for testing therapies to extend healthy aging and longevity. The C57BL/6J strain is the most used of all inbred mouse strains. With an average lifespan of about 28 months, exceptionally reaching 36 months [1,2], C57BL/6J mice allow the development of longitudinal studies on longevity, which are harder to perform in humans. Moreover, several biomarkers of aging, measurable parameters associated with age-related changes, have been identified and validated in mice, like those used for humans. These include cellular and molecular markers based on laboratory measurements as well as phenotypic and functional parameters [3,4]. Such biomarkers quantify the biological and functional age of an individual and can be a valuable tool to predict lifespan and health span.

Among molecular biomarkers of aging, epigenetic clocks are considered one of the most reliable [5,6]. Based on the quantification of methylated cytosine residues in CG dinucleotides (CpGs), epigenetic clocks measure specific age-associated DNA methylation profiles. During aging, there is an overall decrease in DNA methylation, especially in regions lacking coding genes (about 50% of the genome), which can lead to abnormal expression of retrotransposable elements (highly abundant in these regions) [7]. In addition, there is increased methylation of some genes that are important for the oxidative stress response and DNA damage repair, which may contribute to the accumulation of cellular damage and the onset of age-related diseases [8]. In humans, epigenetic clocks have been shown to accurately estimate chronological and biological age and predict survival [9,10,11,12]. In mice, a three CpG clock, analyzing methylation sites in Prima1, Hsf4 and Kcns1 genes, was recently demonstrated to be highly correlated with chronological age [13,14]. This kind of epigenetic clock provides exceptional advantages in terms of time and costs, especially in longitudinal studies involving hundreds of mice. However, the relationship between the three CpG clock, health span and longevity has yet to be investigated.

From a phenotypic point of view, aging is well described by the concept of frailty, defined as a condition of high vulnerability to adverse health outcomes [15]. The Clinical Frailty Index, a measure of frailty based on a clinical evaluation of health deficits, tightly correlates with the risk of disease and death, and is used to predict biological age and survival, in humans as well as in mice [16,17]. A recent longitudinal study on a large cohort of mice developed a novel frailty assessment tool, named the Vitality Score (VS), which combines the Clinical Frailty Index (CFI) with a new Physical Function Score (PFS), a continuous measure of the overall physical performance summarizing the five Fried’s areas of frailty (shrinkness, weakness, endurance, slowness and activity) [2]. The combined VS inversely correlates with chronological age, declines as death approaches, predicts mortality and shows a negative correlation with biomarkers of aging, including the epigenetic clock, thus providing an overall evaluation of the health status [2].

In a previous study, the progression of frailty in aging mice was shown to be delayed by the delivery of the human Longevity-Associated Variant (LAV) of the Bactericidal/Permeability-Increasing Fold-Containing Family B member 4 (BPIFB4) gene through an adenovirus viral vector [18]. The BPIFB4 protein, implicated in activating homeostatic processes such as adaptive stress response and proteostasis, is found at high levels in the serum of long-living people and the LAV-BPIFB4 genetic variant has been associated with healthy aging and exceptional longevity [19]. In addition, the LAV-BPIFB4 gene therapy in mouse models has been proven to rescue the age-related endothelial dysfunction [20], reduce the progression of cardiovascular diseases [21], delay heart aging [22], and decrease the pool of senescent cells and senescence-associated inflammation [23,24]. In such pre-clinical studies, the LAV-BPIFB4 gene was delivered through an adeno-associated virus (AAV serotype 9) carrying a liver-specific promoter. Those studies demonstrated that the liver is the main target of AAV-LAV-BPIFB4 infection, which is also followed by (i) a rapid rise in the plasma level of the BPIFB4 protein [25], (ii) BPIFB4 enrichment in CD11b+ myeloid cells both in bone marrow and in blood without any mRNA upregulation (either human or murine), (iii) over-expression of the human protein in most tissues of transfected mice, such as the aorta and heart [22,25,26], and an increase in BPIFB4 in myocytes and vascular cells [22]. Collectively, previous data suggest that (i) the liver was transduced by the viral vector and (ii) the upregulation in vessels or immune cells of LAV-BPIFB4-treated mice was likely due to an uptake of circulating protein and not to an induction of the endogenous murine gene. At the molecular level, the stable expression of the LAV-BPIFB4 isoform in cultured neurons was shown to reduce DNA damage and to impact epigenetic histone modification and consequent chromatin condensation through the reduction of methylated histone 3 levels [27].

In the present study, we investigated the effect of the systemic gene transfer of human LAV-BPIFB4 in geriatric C57BL/6J mice on epigenetic mechanisms associated with aging through the three CpG clock methylation analysis [13] and the calculation of its ticking rate [28]. Moreover, we combined the methylation analysis with the frailty assessment, to investigate the relationship between epigenetic age, clinical and physical decline, and longevity.

## 2. Results

**Figure 1:**
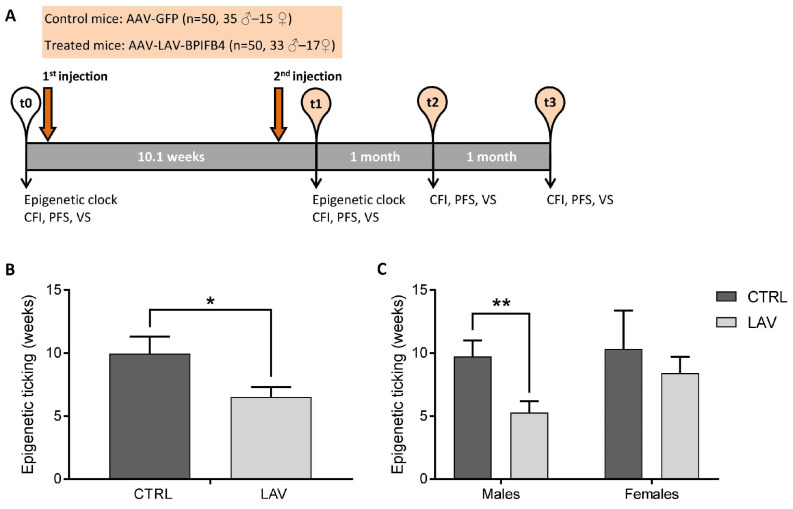
Effect of AAV-LAV-BPIFB4 on the epigenetic ticking rate in mice. (**A**) Schematic representation of the experimental design. (**B**) Epigenetic ticking rate in control and treated mice. (**C**) Epigenetic ticking rate in control and treated mice subdivided based on sex. Values are obtained by calculating the difference of epigenetic age (in weeks) between the t1 and t0 time-point. Values are expressed as means ± SEM. Statistics to compare epigenetic ticking between the treatment and control group were performed using mixed model analysis for longitudinal data (SPSS 26.0), including treatment and sex as fixed factors; * *p* < 0.05; ** *p* < 0.01. BPIFB4 = Bactericidal/Permeability-Increasing Fold-Containing Family B member 4; LAV = Longevity-Associated Variant; GFP = green fluorescent protein; CFI = Clinical Frailty Index; PFS = Physical Function Score; and VS = Vitality Score.

The epigenetic age was measured through the three CpG clock method [13] in blood cells of aged mice before (time 0, t0; age: 19.2–26.6 months) and after (time 1, t1; age: 21.5–29 months) two i.v. injections of LAV-BPIFB4 or GFP (control) -encoding adeno-associated viral vectors (AAV) (Figure 1A). The epigenetic ticking, i.e., the difference of the epigenetic age between the t1 and the t0, was then calculated for each mouse. The gene therapy with LAV-BPIFB4 resulted in a significant reduction of epigenetic ticking. Control mice showed an average epigenetic ticking of 9.963 ± 1.358 weeks, comparable with the difference in chronological age between the pre- and post-treatment period (10.1 weeks), while in mice injected with LAV-BPIFB4, the average epigenetic ticking was reduced to 6.526 ± 0.780 weeks (Figure 1A). Such effect was predominant in males and not significant in females (Figure 1B). With an average ticking value of 5.292 ± 0.898 weeks, the epigenetic aging was slowed down by more than four weeks in males by the systemic treatment with LAV-BPIFB4.

**Figure 2:**
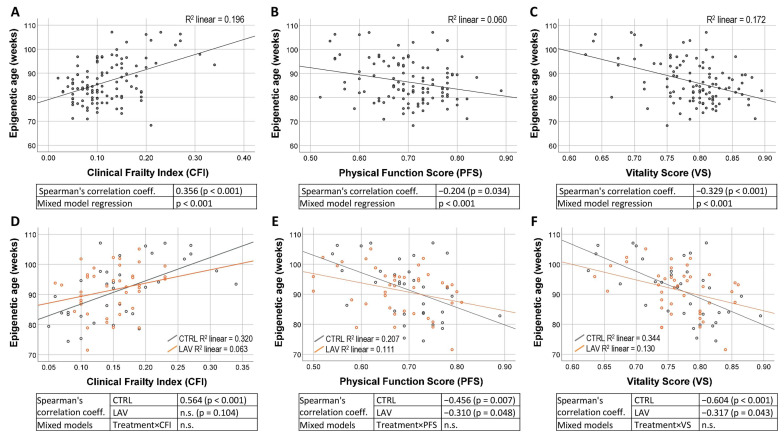
Correlation between the epigenetic age and the frailty scores. (**A**–**C**) Relationship between epigenetic age and CFI (**A**), PFS (**B**) and VS (**C**) evaluated in untreated mice (all mice at t0 and control mice at t1) using Spearman’s correlation and mixed model analysis for repeated measures. The regression equations estimated through the mixed model are reported: y = 70.2x + 78.2 for CFI (**A**), y = −29.0x + 106.7 for PFS (**B**) and y = −68.4x + 140.5 for VS (**C**). (**D**–**F**) Correlation between the epigenetic age and CFI (**D**), PSF (**E**) or VS (**F**) in control (CTRL) and treated (LAV) mice in the post-treatment period (t1). The R^2^ coefficients are shown on each graph. Correlation coefficients and p values analyzed by Spearman’s correlation and mixed models are indicated. n.s. = not significant.

Since we previously demonstrated (i) a delay in the progression of frailty in LAV-BPIFB4-AAV-injected old mice [18] and (ii) a correlation between the 3 CpG epigenetic age and the frailty scores in C57BL6/J mice [2], we investigated whether and at what extent the LAV-BPIFB4 gene therapy may affect the relationship between epigenetic age and frailty. Therefore, the frailty scores (CFI, PFS and VS) were recorded at t0 and post-treatment, with monthly frequency from t1 to t3 (Figure 1A). We first verified the association between epigenetic age and frailty parameters in our study population of untreated mice, confirming a positive correlation with CFI (Spearman’s corr. coeff. = 0.356; *p* < 0.001) and a negative correlation with PFS (corr. coeff. = −0.204; *p* < 0.034) and VS (corr. coeff. = −0.329; *p* < 0.001) (Figure 2A–C), as previously demonstrated [2]. The regression analysis estimated that the epigenetic age increases by 7 weeks for each 0.1 increase in CFI, by 2.9 weeks for each 0.1 decrease in PFS and by 6.8 weeks for each 0.1 decrease in VS (Figure 2A–C). We then compared control and treated mice in the post-treatment t1 period. In both control and treated mice, the epigenetic age correlated with almost all analyzed parameters (Figure 2D–F). A comparison between the slopes of control and treated group (Figure 2D–F) through a mixed model interaction analysis did not evidence significant differences, suggesting that the LAV treatment does not significantly influence the association between epigenetic age and the frailty scores. The correlations between the epigenetic age and the frailty scores at t1 were then assessed by separating animals on the basis of their sex (Figure S1). A generalized loss of significant correlations was observed in both males (Figure S1a–c) and females (Figure S1d–f) compared to those performed without separating by sex, likely due to the reduced sample size.

To investigate the association between the epigenetic ticking rate with the frailty increasing speed, we calculated ΔCFI, ΔPFS and ΔVS as the difference of CFI, PFS and VS, respectively, between the post-treatment (t1, t3 and t3) and the pre-treatment period, for each mouse. Such longitudinal measurements may allow an understanding of how fast the individuals aged in a selected time frame at epigenetic, clinical and functional levels and the influence of the treatment on such aging rates. First, we verified with the mixed model analysis of the epigenetic ticking that the interactions between treatment and delta frailty scores (ΔCFI, ΔPFS and ΔVS) were not significant, meaning that the relationship between epigenetic changes and functional changes is retained independently of the treatment. Therefore, we studied the interaction between these variables without separating the treatment groups. In females, the relationship between epigenetic ticking and the frailty scores was significant only at t2 (Table 1). In males, ΔCFI was significantly related to the epigenetic ticking only at t1, ΔPFS at t2 and t3, and ΔVS at all time periods (Table 1).

**Figure 3:**
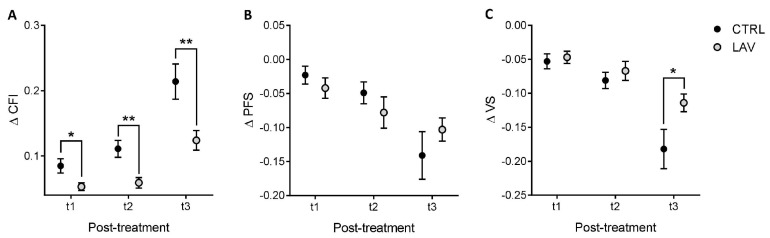
Effect of AAV-LAV-BPIFB4 on the longitudinal variation in the frailty scores in old male mice. (**A**–**C**) Longitudinal variation in CFI (**A**), PFS (**B**) and VS (**C**) in the subgroup of old male mice (age at t0 > 20 months) over the different post-treatment periods (t1, t2 or t3). Values of ΔCFI, ΔPFS and ΔVS are obtained by calculating the difference of the respective score between the post-treatment (t1, t2 or t3) and the t0 time-point. Values are expressed as means ± SEM. Statistics to compare ΔCFI, ΔPFS and ΔVS between the treatment and control group were performed using mixed model analysis for longitudinal data (SPSS 26.0); * *p* < 0.05 and ** *p* < 0.01.

The average ΔCFI, ΔPFS and ΔVS did not show significant differences in the whole population between control and treated group, not even when calculated over a longer time-period. However, the analysis of a subgroup of older mice (age at t0 > 20 months) revealed that in males the average ΔCFI was significantly decreased by the AAV-LAV-BPIFB4 injection in the post-treatment period, with major effects at t2 and t3 (*p* < 0.01) (Figure 3A). ΔPFS did not show any significant variation over time, although an increasing trend of LAV-treated animals compared to controls was observed at the last time-point (t3), while the ΔVS values were significantly higher in treated mice at t3 (Figure 3B,C).

## 3. Discussion

Discovered as a peculiarity of centenarians and long-living people, the LAV-BPIFB4 protein turned out to be a very promising therapeutic strategy to fight the aging processes. The “rejuvenating” properties of the LAV-BPIFB4 were demonstrated in pre-clinical models, with a broad spectrum of protective effects towards different pathological conditions, including inflammatory status [23], cardiovascular problems [21] and Huntington’s disease [27]. Interestingly, recent evidence demonstrated that LAV delivery caused a rejuvenation of the hearts of old mice by a human equivalent of more than ten years [22]. Here, we demonstrated that the LAV gene therapy was also able to slow down the epigenetic age progression in nucleated blood cells of aged mice.

The genome undergoes a global hypomethylation as age progresses, leading to chromatin remodeling and the activation of genes that contribute to the aging process. However, DNA methylation is a reversible modification, which can be reverted by environmental factors and external interventions. In this study, measuring the longitudinal variation in the epigenetic clock allowed us to determine the impact of the LAV treatment on DNA methylation patterns. The epigenetic clock in the blood cells of control mice moved forward by about ten weeks, matching the chronological distance between the two blood sample collections, and further confirming the accuracy of the 3 CpG methylation analysis for mice. After two months since the first injection, the systemic gene therapy with the LAV-BPIFB4 variant elicited a clear deceleration of the epigenetic age in the blood of old mice, reducing the epigenetic ticking by more than three weeks (four weeks considering only males), which is equivalent to two to three years in humans. Although such a reduction was more evident in males, female mice showed the same trend, and the absence of a significant difference may be likely due to the smaller sample size compared to the male one. In support of a sex-independent action of the LAV therapy, its effect on the cardiac function was clearly demonstrated in both male and female mice [22]. Nonetheless, a sex-specific activity was previously ascribed to the BPIFB4 protein, which inversely correlated with the severity of the COVID-19 disease in men but not in women [29]. Moreover, the gender effect in the LAV gene therapy could also be explained by a higher efficiency of AAV transduction in males, as previously described in C57BL/6 mice injected with AAV constructs, demonstrating that the transgene expression was two- to three-fold higher in males than in females [30].

Our observation of a reduced rate of epigenetic aging in blood cells corroborates the rejuvenating effect of the LAV gene therapy on the immune system of old mice, demonstrated by decreased senescence among the peripheral blood mononuclear cells, restored NAD^+^ plasmatic levels and reduced CD38^+^ macrophages after 60 days of AAV-LAV-BPIFB4 infection [23]. Similar to our observation, these effects on the blood immune cells were detected in males.

The epigenetic rejuvenation of blood cells may be related to the ability of the LAV-BPIFB4 protein to reduce the inflammatory status as highlighted in pre-clinical models, both in vitro (cultured monocytes, dendritic cells and pericytes [22,31]) and in vivo (AAV-LAV-BPIFB4 treated mice [23]), and in long-living individuals [31,32]. Indeed, a link between chronic low-grade inflammation, another prominent feature of aged individuals, and epigenetic changes has recently emerged. Association studies identified several DNA methylation sites associated with inflammatory markers, e.g., in human leukocytes [33], and suggestive of a global hypomethylation [34]. However, since the causality relationship between inflammation and epigenetic changes in aging is still to be elucidated (whether the pro-inflammatory environment induces epigenetic aging, or age-related epigenetic modifications dysregulate inflammatory pathways), we can only speculate if the LAV effect on epigenetic aging is a direct action or a consequence of the reduced inflammation. Nevertheless, a direct impact of the LAV therapy on epigenetic modifications is not to be excluded, given that LAV-BPIFB4 showed a nuclear localization [35] and an effect on histone-mediated chromatin remodeling has been previously demonstrated [27].

Several anti-aging treatments are suggested to have an underlying epigenetic mechanism. Indeed, a reduction of epigenetic age was observed in several murine tissues after different intervention typologies, including rapamycin, dasatinib + quercitin, caloric restriction and exercise [36,37].

Among the various effects observed after the LAV-BPIFB4 systemic gene delivery, the delayed occurrence of frailty [18] can be of particular interest for the potential of this treatment to improve the health span, since frailty is a known risk factor for several diseases and mortality [38]. Thus, delaying of the frailty onset may be strategic for preventing other pathological conditions. Our data confirmed a protective role of the LAV therapy towards the progression of clinical frailty in aged animals, and supported a significant benefit in the older, as previously observed [18]. Moreover, we demonstrated that the relationship between epigenetic and functional changes is independent of the treatment, consistent with an effect of the treatment on both parameters. We further explained that the LAV therapy positively affects the Vitality Score, corroborating this tool as a reliable predictor of global health status [2]. The delayed effect on the Vitality Score, highlighted two months after the impact on DNA methylation, supports the concept of a non-synchronicity between molecular and functional outcomes [3]. Indeed, the epigenetic ticking (measured at t1) was more related to later variations of the frailty status (measured at t2 and t3), both in males and in females, confirming that biological changes precede functional ones [39]. As previously discussed for epigenetic variations, the anti-inflammatory action of the LAV-BPIFB4 protein may also be the key to the delay of frailty onset, since low-grade chronic inflammation is considered to play a role in frailty [40]. It is conceivable that the conditioning effects of circulating protein may favorably skew the inflammatory burden and improve endothelial cells both at the peripheral site and CNS, thus ameliorating the overall frailty condition.

A limitation of this study is the use of the mice three CpG clock method, which differs from the methods implemented in humans; these methods analyze hundreds of CpG dinucleotides. Although the analysis of three CpG dinucleotides may be subjected to some variability, the technique was proven to have a similar level of accuracy to existing models, but with lower costs and execution times [13]. In addition, our observation of epigenetic rejuvenation is limited to blood, but it is known that the epigenetic aging may progress at different rates in different cell types. Therefore, future studies on other tissues would help clarify the role of epigenetic mechanisms in LAV-BPIFB4 protective action. However, blood remains one of the less invasive and more easily accessible tissues.

In conclusion, our finding suggests that LAV-BPIFB4 gene therapy may delay the aging-related epigenetic modification in aged mice, in association with an improving of the frailty condition. This confirms the potential of such proteins for future treatments to prevent human frailty.

## 4. Materials and Methods

### 4.1. Experimental Design

The animal study was performed according to the European Community Council Directives of 2010/63/UE, and the experimental protocol was approved according to the current Italian law (D.Lgs. n. 26/2014) by the Organismo Preposto al Benessere Animale (OPBA, animal care and health committee) of IRCCS INRCA and by the General Direction of Animal Health and Veterinary Drugs of the Italian Ministry of Health with authorization n° 130/2018-PR. 

C57BL/6J mice were housed in a specific pathogen-free (SPF) facility, under a controlled temperature (22 ± 2 °C), with a 12 h light–dark cycle and ad libitum access to food and water. The study was conducted as described [18] and the experimental plan is summarized in Figure 1A. A total of 100 mice (68 males and 32 females) were used. The mice were assigned to two age-matched experimental groups: a treatment group (AAV-LAV-BPIFB4; 50 mice: 33 males and 17 females) and a control group (AAV-GFP; 50 mice: 35 males and 15 females). The adeno-associated viral vectors (AAV serotype 9 with a TBG promoter) used to deliver the LAV-BPIFB4 and the control protein were produced as previously described [20]. At the start of the study (t0), the mice were aged between 19.2 and 26.6 months. A total of 1 × 10^14^ viral particles of AAV-LAV-BPIFB4 or AAV-GFP were intravenously (i.v.) injected in the tail vein of the treatment and control animals, respectively. A second i.v. injection was performed after two months to ensure a sustained therapeutic effect over time. Blood samples from each animal were collected before the treatment (t0) and 10.1 weeks after the treatment (t1) and stored at −80 °C until their use for epigenetic clock analyses. A non-invasive clinical and physical frailty assessment was performed before the treatment (t0) and post-treatment, with monthly frequency, three times (t1, t2 and t3). Mice were maintained and observed until natural death or euthanasia was practiced for humane reasons. 

### 4.2. Epigenetic Clock Analysis 

DNA methylation analysis was performed using the three CpG method as previously described [13]. Genomic DNA was isolated from 200 µL whole blood using the QIAamp DNA Blood Mini Kit (Qiagen, Hilden, German), and the concentration of DNA samples was determined using the NanoDrop 2000 Spectrophotometer. For each sample, 500 ng of genomic DNA were used for the bisulfite conversion (unmethylated cytosines were converted into uracil bases). Bisulfite conversion was performed with the Epitect Fast DNA Bisulfite kit (Qiagen), according to the manufacturer’s instructions. Next, 30 µg of converted DNA, quantified using the NanoDrop spectrophotometer, were amplified by PCR using methylation-specific primer pairs for the Prima1, Hsf4 and Kcns1 genes (Table S1) using the PyroMark PCR Kit (Qiagen). The reverse primer of each pair was biotinylated. The PCR method consisted of one cycle at 95 °C (15 min); 45 cycles at 95 °C (30 s), 56 °C (30 s) and 72 °C (30 s); and one cycle at 72 °C (10 min). A total of 5 µL of the PCR product was immobilized to 2 µL Streptavidin Sepharose High-Performance Bead (Cytiva, Sweden) and then annealed to 20 µL sequencing primer (0.375 μM) (Table S1) for 5 min at 80 °C. Amplicons were sequenced on a pyrosequencing system (PyroMark Q24 System; Qiagen, Hilden, Germany) using PyroMark Q24 Advanced Reagents (Qiagen). The sequences were analyzed with PyroMark Q24 Advanced software (v 3.0.0 from Qiagen) to calculate the methylation level at selected CpG sites (Figure S2). The epigenetic age was calculated through the equation described in [13] and using the coefficients obtained in [2]. The epigenetic ticking rate was calculated as the difference of the epigenetic age values between t1 and t0.

### 4.3. Measurement of Clinical Frailty Index, Physical Function Score and Vitality Score 

The Clinical Frailty Index (CFI) and the Physical Function Score (PFS) were measured in mice as previously described [2,18,41]. All frailty measurements were performed within the SPF animal facility of INRCA in a dedicated area. The CFI score for each mouse was calculated using the previously published 31 items checklist and method [41]. The measurement of the PFS in mice was performed following the same procedure described in [2,17]. Briefly, the score was obtained by combining the measurement obtained from the five criteria of the frailty assessment (shrinking, weakness, exhaustion, slowness and sedentarily), in order to obtain a continuous value ranging from 1 (optimal health) to 0. To ensure testing reliability, we adapted the mice to the tests for two months before the start of the study, and we performed multiple measurements for each of the five criteria. Details on procedures and calculations can be found in [2]. For the Vitality Score (VS) calculation, a Clinical Health Score (CHS) was first computed as 1-CFI. This value ranged from 1 (optimal health) to 0. The VS was obtained by calculating the arithmetic mean of the individual values of CHS and PFS. The VS score ranged from 1 (optimal health) to 0 [2]. 

Values of ΔCFI, ΔPFS and ΔVS were obtained by calculating the difference of the respective score between the post-treatment (t1, t2 or t3) and the t0 time-point.

### 4.4. Statistical Analysis

The significance of differences between the control and treated animals was tested through the generalized linear mixed model analysis (SPSS 26.0) to consider the longitudinal design of the study. The linear model was developed assuming a gamma distribution. The Satterthwaite approximation and robust estimator were used to account for unbalanced data and violation of the assumptions. The regression analysis for repeated measures was performed through the linear mixed models tool using epigenetic age as the dependent variable and the frailty scores as the covariate and random effect. We also selected the intercept and maximum likelihood method. The correlations between epigenetic age and frailty scores were analyzed using Spearman’s correlation (SPSS 26.0). To compare the slopes of the control and treated groups, an interaction item was added between the treatment and frailty scores in the mixed model. The relationships between epigenetic ticking and delta frailty scores (ΔCFI, ΔPFS and ΔVS) were assessed using the mixed model analysis.

## Figures and Tables

**Table 1:** Relationship between the epigenetic ticking rate and the longitudinal variation in the frailty scores (ΔCFI, ΔPFS and ΔVS) in males and females. Values of ΔCFI, ΔPFS and ΔVS are obtained by calculating the difference of the respective score between the post-treatment (t1, t2 or t3) and the t0 time-point.

	Females		Males	
	F	*p*	F	*p*
ΔCFI (t1 − t0)	1.143	0.145	4.124	0.048
ΔCFI (t2 − t0)	6.019	0.020	1.879	0.177
ΔCFI (t3 − t0)	0.295	0.592	1.372	0.248
ΔPFS (t1 − t0)	0.834	0.368	2.966	0.091
ΔPFS (t2 − t0)	13.235	0.001	6.689	0.013
ΔPFS (t3 − t0)	0.001	0.991	10.210	0.003
ΔVS (t1 − t0)	1.378	0.250	5.221	0.027
ΔVS (t2 − t0)	13.146	0.001	6.098	0.017
ΔVS (t3 − t0)	0.036	0.852	5.117	0.029

## Data Availability

The data that support the findings of this study are available from the corresponding author (MM) upon reasonable request.

## Supplementary Materials

The following supporting information can be downloaded at https://www.mdpi.com/article/10.3390/ijms24076464/s1.
