# Supplementary material for: Effects of Human LAV-BPIFB4 Gene Therapy on the Epigenetic Clock and Health of Aged Mice
Source: Int J Mol Sci. 2023 Mar 30;24(7):6464. doi: 10.3390/ijms24076464 (PMC10095240; doi:10.3390/ijms24076464)
Supplement: Supplementary file 1 [file ijms-24-06464-s001.zip › ijms-2282792-supplementary.pdf]

Table S1. Nucleotide sequences of the forward, reverse and sequencing primers for the Prima1, Hsf4 and Kcns1 genes, used for the three-CpG clock analysis

Gene	Primer	Sequence
**Prima1**	Forward	5'- AGGAGAGGTAAATTATGAATTAGGTTTATA -3'
	Reverse	5'-Biotin- TCTTACTCAAACCTCCCTATAATCTTTTC -3'
	Sequencing	5'-AATTATGAATTAGGTTTATATTTT-3'
**Hsf4**	Forward	5'- GGATGGTGTTTTTTGTTTGTAGGTA -3'
	Reverse	5'-Biotin- ACTTACACCTCTCCCAACA -3'
	Sequencing	5'- GTGTTTTTTGTTTGTAGGTAT-3'
**Kcns1**	Forward	5'- GTGTTGGGAGTTAGTAGTAGG -3'
	Reverse	5'-Biotin- ACCATATACATCCACAACCTACC -3'
	Sequencing	5'- GATATTTAGAAGTTGAATTAAG -3'

Figure S1. Correlation between epigenetic age and frailty scores in males and females. Correlation between the epigenetic age and CFI (a, d), PSF (b,e), or VS (c,f) in control (CTRL) and treated (LAV) mice in the post-treatment period (t1) in males **(a-c)** and females **(d-f)**. The R^2 coefficients are shown on each graph. Correlation coefficients and p values analyzed by Spearman's correlation are indicated. n.s. = not significant.

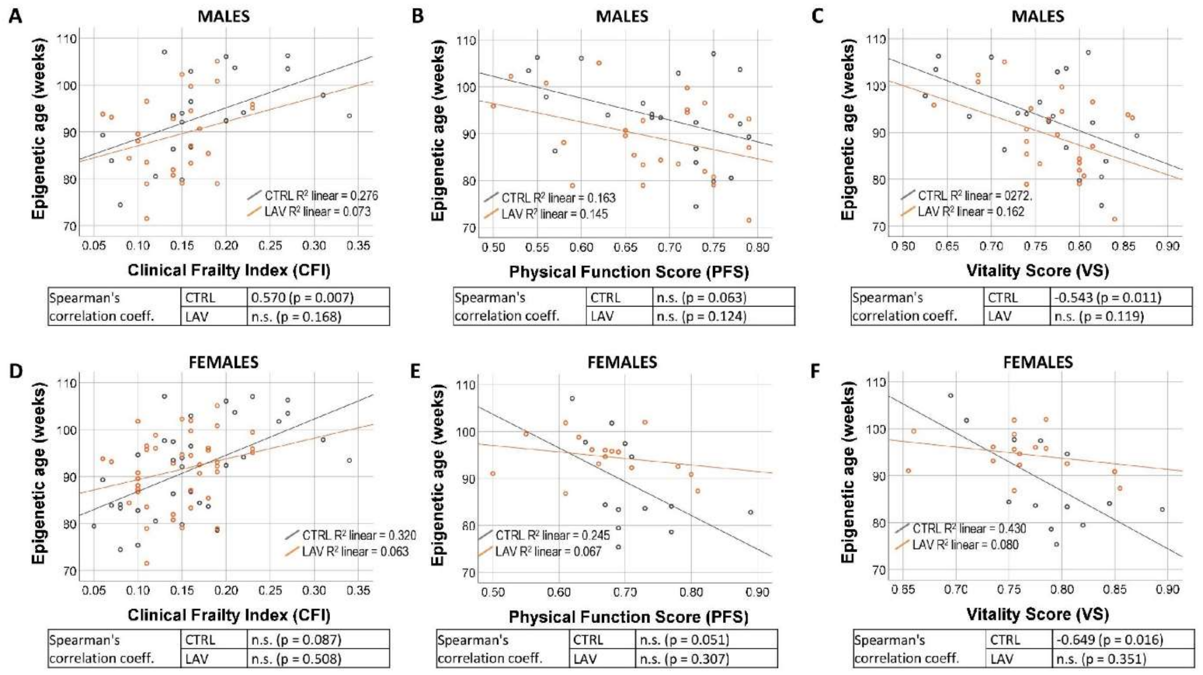

Figure S2. Target sequences of the sequencing for the three-CpG clock analysis. CpG sites are highlighted.

Prima1

5'-AATTATGAATTAGGTTTATATTTTT**CG**GGTGGGGGGTAA**CG**A**CG**G-3'

1 2 3

Hsf4

5'-GTGTTTTTTGTTTGTAGGTATTTG**CGT**TG**CG**AGG**CG**ATGATAGT**CG**ATGG**CG**TT**CG**GAAGATTTGAGC**CG**-3'

1 2 3 4 5 6 7

Kcns1

5'-GATATTTCGAAGTTGAATTAAG**CG**ATGTAGAAGTATTTTAGG**CG**G**CG**TAGTAT**CG**-3’

1 2 3 4
